# Exploring the Influence of the Coronavirus Disease 2019 Pandemic on the Accessibility of Rehabilitation Services Provided to Children with Disabilities: A Cross-Sectional Study

**DOI:** 10.3390/medicina59050837

**Published:** 2023-04-26

**Authors:** Safaa Mostafa Elkholi, Monira I. Aldhahi, Nisreen Naser Al Awaji

**Affiliations:** 1 Department of Rehabilitation Sciences, College of Health and Rehabilitation Sciences, Princess Nourah bint Abdulrahman University, P.O. Box 84428, Riyadh 11671, Saudi Arabia; smelkholi@pnu.edu.sa; 2 Department of Health Communication Sciences, College of Health and Rehabilitation Sciences, Princess Nourah bint Abdulrahman University, P.O. Box 84428, Riyadh 11671, Saudi Arabia; nnalawaji@pnu.edu.sa

**Keywords:** children with disabilities, COVID-19, occupational therapy, physical therapy, rehabilitation, speech therapy

## Abstract

*Background and Objectives*: Children with disabilities (such as cerebral palsy, autistic spectrum disorder, and Down syndrome) are the most vulnerable and marginalized subset of the population, representing 2.7% of the total population of Saudi Arabia. The COVID-19 outbreak might have disproportionately affected children with disabilities, augmented their isolation, and induced severe disruptions to the services on which these children rely. Limited research has been conducted in Saudi Arabia to understand the impact of the COVID-19 pandemic on the rehabilitation services provided to children with disabilities and barriers. This study aimed to investigate the effect of the lockdown implemented as a result of the coronavirus disease-2019 (COVID-19) pandemic on the accessibility of rehabilitation services, including communication, occupational therapy, and physical therapy, in Riyadh, Kingdom of Saudi Arabia. *Materials and Methods*: In this cross-sectional study, a survey was conducted between June and September 2020 during the lockdown in Saudi Arabia. A total of 316 caregivers of children with disabilities from Riyadh participated in the study. The accessibility of rehabilitation services provided to children with disabilities was assessed by designing a valid questionnaire. *Results*: A total of 280 children with disabilities received rehabilitation services before the COVID-19 pandemic and showed improvement following therapeutic sessions. However, during the pandemic, most children stopped receiving therapeutic sessions because of lockdown, which deteriorated their condition. This shows a significant reduction in the accessibility of the rehabilitation services provided during the pandemic. *Conclusions*: The findings of this study revealed a drastic decline in services provided to children with disabilities. This caused a notable deterioration in the abilities of these children.

## 1. Introduction

Rehabilitation services are a fundamental component of the high-value care offered to children with disabilities to optimize their physical, psychological, and cognitive functioning [1]. The unprecedented outbreak of the novel coronavirus disease 2019 (COVID-19) has led to staggering demands in all life sectors, including rehabilitation services [2]. The rapid spread of the infection, the expanding number of cases, and related mortality have caused the government to take many protective measures [3]. These measures included the implementation of quarantine, restrictions on travel and development activities, the cancellation of social events, and the suspension of many other services [2]. The pandemic prevention measures in Saudi Arabia are comprehensive, as the country has enforced mandatory social distancing, quarantine, and school closures [2,3]. Consequently, certain groups of the population, such as children with disabilities, are affected more significantly. Inevitably, to abide by the government’s orders, some rehabilitation centers had to either suspend their operations or partially operate at reduced capacity. It is unknown if decisions meant for the protection of both children with disabilities and healthcare workers have impacted the rehabilitation services provided to these children and contributed to activity limitations secondary to the lack of necessary rehabilitation care. Lockdowns might have magnified the needs of children with disabilities, as their parents had to deal with different strategies to cope with home-based interventions.

Children with disabilities (such as cerebral palsy, autism spectrum disorder, and Down syndrome) are the most vulnerable and marginalized subset of the population, representing 2.7% of the total population of Saudi Arabia [4]. These children have several complications ranging from medical problems such as epilepsy, spasticity, and hypotonicity to developmental and functional impairment. Therefore, rehabilitation is an essential longitudinal process that reduces impairments, improves quality of life and health status, and enhances the independence of these children and their inclusion within the community. The COVID-19 outbreak might have disproportionately affected children with disabilities, augmented their isolation, and induced severe disruptions to the services on which these children rely. The COVID-19 pandemic has had a significant impact on the accessibility of rehabilitation services for children with disabilities. The pandemic has disrupted healthcare systems and created new challenges for families seeking care for their children. As a result, many children with disabilities may have experienced a decrease in the availability and quality of rehabilitation services. Consequently, home-based exercises or telerehabilitation have been encouraged [5]. Family plays an essential role in the rehabilitation process of children; however, it is also unknown whether parents can convey the rehabilitation program to their children at home and cope with the current situation. Despite a growing body of evidence indicating that disability is a crucial social and economic issue, limited research has been conducted in Saudi Arabia to understand the impact of the COVID-19 pandemic on the rehabilitation services provided to children with disabilities and barriers. Therefore, the overarching aim of this study is to assess the proportion of changes in the accessibility of services provided to children with disabilities before and during the COVID-19 pandemic. To investigate the extent to which rehabilitation services have been challenged by the pandemic. A comprehensive understanding of the needs and services offered to children with disabilities and the potential factors that hinder their participation is essential for stakeholders and healthcare professionals who contribute to delivering effective services for these children.

## 2. Materials and Methods

### 2.1. Study Design and Participants

A cross-sectional study was conducted between June and September 2020, during the COVID-19 pandemic in Riyadh. The study adopted a quantitative approach in which caregivers of children with disabilities completed a self-administered questionnaire. The questionnaire was designed to assess the rehabilitation services provided in Riyadh, including physical, occupational, and speech therapy, and to investigate the extent of barriers to these rehabilitation services during the COVID-19 pandemic. Five experts revised the questionnaire to measure its validity, and a pilot test was conducted by distributing the questionnaire to 30 caregivers to determine its reliability.

After pilot testing, a link to the online questionnaire was distributed via email to all rehabilitation centers and different support groups comprising caregivers of children with disabilities in Riyadh. A sample size of 340 children with disabilities was estimated for this study based on the prevalence of children with disabilities in Riyadh [6], with a confidence level of 95%, a margin of error of 5%, and a study power of 0.8. 

In this study, 316 caregivers of children with disabilities voluntarily participated by completing an online questionnaire. Prior to answering the questionnaire questions, participants were required to provide their agreement to participate through a consent form that included a description of the study’s aim and target population. Participation in the online questionnaire was voluntary, and no remuneration was paid for participation. 

### 2.2. Inclusion Criteria 

Caregivers of children with disabilities (such as those diagnosed with physical, mental, speech, or communication disorders) were invited to participate in the study based on the following criteria: age 1–18 years and living in Riyadh.

### 2.3. Procedure 

An online questionnaire was generated in Arabic using Google Forms. The questionnaire was validated by five experts, who were chosen for their experience in the rehabilitation field. They judged the questionnaire and requested some modifications that were made by the authors. The experts confirmed that the questionnaire had face, content, and validity. Subsequently, the initial draft of the questionnaire was distributed to a pilot sample of 30 caregivers to assess its reliability. The data were tested for internal consistency, and Cronbach’s coefficient alpha was 0.85, indicating high internal consistency. 

The questionnaire included three sections. The first section includes personal and demographic information and consists of two parts. The first part included data on caregivers, such as their place of residence, relation to the child, number of siblings, socioeconomic status, and education level. The second part involved questions about the child with a disability, including age, sex, type of impairment(s), and diagnosis if any of the siblings had a disability.

The second section addressed the accessibility of rehabilitation services before the COVID-19 pandemic. It included questions about the type of rehabilitation services provided if the caregiver was a member of a support group, the abilities and independence level of the child, satisfaction level with rehabilitation services using the Likert scale, and improvements noted in the abilities of the child following rehabilitation sessions. 

The third section addressed the accessibility of rehabilitation services after the COVID-19 pandemic. It consisted of questions about the effect of the lockdown on the accessibility of rehabilitation services during the lockdown, barriers to accessibility, intention to resume sessions, type, duration, and efficacy of rehabilitation sessions during the lockdown, barriers to the application of home programs, and intention to resume therapeutic sessions in rehabilitation centers.

### 2.4. Ethical Considerations

This study was approved by the Institutional Review Board of Princess Nourah bint Abdulrahman University on 16 June 2020 (IRB:20-0247), Riyadh, Saudi Arabia. The procedures of the study and rights of the respondents were explained at the beginning of the questionnaire, and written informed consent was obtained from all respondents before participation.

### 2.5. Statistical Analysis

All data collected were screened to identify the presence of missing data, outliers, and normality. In the process of data cleaning, no missing data were found. The Shapiro–Wilk test was performed to test the normality assumption of the data. Descriptive statistics (frequencies and percentages) were used to present the demographic and categorical data of the participants. Median values were calculated for the numerical data. Cronbach’s alpha was used to measure internal consistency (reliability). McNemar’s test was conducted to compare the accessibility of the rehabilitation services provided before and during the COVID-19 pandemic. The Kruskal–Wallis test was carried out to compare satisfaction with and benefits of services across different disabilities. All statistical analyses were performed using SPSS Statistics for Windows (version 25; IBM Corp., Armonk, NY, USA).

## 3. Results

### 3.1. Participant Characteristics

A total of 316 caregivers of disabled children participated in this study. Of the total, 29.4% of the caregivers reported having a child between the ages of four and six years, and the majority (50.3%) were males from Riyadh. The respondents’ caregivers voluntarily participated in the study. The socio-demographic characteristics of the children are presented in Table 1.

### 3.2. Therapeutic Services Received by the Children of the Study Group before the COVID-19 Pandemic

As presented in Table 2, the majority of children (88.6%) received rehabilitation sessions before the pandemic (speech or language, occupational, and physical therapy). Parents’ satisfaction with the therapy services offered was reported to be good. The median value of satisfaction for all therapy services, including speech or language and occupational and physical therapy, was 4 (good). In addition, caregivers’ satisfaction with improvement in their children with therapy services was good, with a mean value of 4 (good) for all speech or language, occupational, and physical therapy (Table 3). 

### 3.3. Therapeutic Services during the COVID-19 Lockdown

Most children have ceased therapy during the COVID-19 pandemic. A total of 230 parents (72.8%) reported that their children’s conditions were delayed after staying at home for long periods and dropping out of the sessions. Most of the participants (63.2 [70.6]) either did not receive any therapeutic services during the COVID-19 pandemic or experienced difficulties in receiving these services (Table 4). On the other hand, only 19 (6%) of the children in the study group continued receiving therapy. They received therapeutic services through telerehabilitation and internet/media services. Some received home sessions from specialists (Table 5). The benefits of the support provided by the specialist during the lockdown period associated with the COVID-19 pandemic were reported as not valuable for speech/language and physiotherapy but valuable to some extent for occupational therapy (Table 5). There was no significant difference in the evaluation of the benefits of offered services (speech, occupational therapy, and physiotherapy) between different diagnoses (*p* > 0.05) (Appendix A).

### 3.4. Comparison of the Accessibility of the Services Provided before and during the COVID-19 Pandemic

There was a significant decrease in the percentage of children who received therapy services during the COVID-19 pandemic compared with those who received these services before the pandemic (*p* < 0.001). Hence, 280 (88.6%) children in the study group received therapy services before the COVID-19 pandemic, whereas only 19 (6%) received therapy services during the COVID-19 pandemic (Table 6).

## 4. Discussion

The COVID-19 pandemic has posed unique challenges to children with disabilities, including reduced access to rehabilitation services. This study assessed the influence of the pandemic on the rehabilitation services provided to children with disabilities and their consequences in terms of access to rehabilitation services. To our knowledge, this is the first study to investigate the influence of the COVID-19 pandemic on children with disabilities and the rehabilitation services provided, as reported by their caregivers. The overarching findings of this study are that most children stopped receiving therapeutic sessions, and their conditions worsened because of the lockdown and closure of rehabilitation centers. Several studies conducted have emphasized the discontinuation of outpatient services due to the lockdown, which has resulted in limited access to outpatient rehabilitation [7].

### 4.1. Satisfaction with Rehabilitation Services

It is important to meet the healthcare needs of a child with a disability, as chronic conditions affect not only the child but also the entire family [8]. Satisfaction with rehabilitation services is also essential for acceptance and adherence to recommended therapeutic regimens. The findings of this study demonstrated that the satisfaction level of caregivers with the rehabilitation services offered to their children before the COVID-19 pandemic was good for all aspects of rehabilitation services, including speech, occupational, and physical therapies. The findings of this study are consistent with a previous report [9] that demonstrated high satisfaction among caregivers of disabled children with the rehabilitation services provided in Malaysia. The present finding is consistent with a study conducted in Saudi Arabia, where parents of disabled children expressed high satisfaction with the speech therapy sessions offered for their disabled children [10]. This supports the notion that speech therapy can be an effective intervention for children with disabilities.

The results of this study showed that caregivers reported an improvement in the communication, physical, and functional abilities of their children following rehabilitation sessions received before the pandemic. This is in agreement with a study conducted by Sechoaro et al. [11], who found that rehabilitation sessions such as occupational and speech therapy were effective in improving the intellectual abilities, communication, and independence in self-care activities of children with mental disabilities. According to a study by Bongo et al. [12], rehabilitation enhances the quality of life of children with disabilities by improving their skills and performance in their environment. Therefore, consistency in providing rehabilitation services is necessary to improve children’s quality of life, enhance their participation and community engagement, and promote their health and wellbeing.

### 4.2. Accessibility of Services before and after the COVID-19 Pandemic

The accessibility of rehabilitation services provided to children with disabilities plays an important role in maintaining a healthy life and boosting the well-being of all individuals with health conditions that affect their functioning [13]. The findings of this study revealed a drastic decline in the services provided to disabled children before and after the COVID-19 pandemic. The results showed that 88.6% of the children received rehabilitation services before the pandemic, and 11.4% did not receive these services. This reveals the availability of rehabilitation services in Saudi Arabia, as the majority of caregivers of disabled children report. A possible reason for not receiving rehabilitation services before the pandemic could be associated with residing in remote areas where access to rehabilitation services is limited. Possible causes of not accessing the services include waiting lists, a lack of knowledge on how to obtain the services, and inadequate awareness of the benefits of these services [14].

However, after the pandemic, 94% of caregivers reported not accessing rehabilitation services. The reason for not having access to these services is not clear; however, several factors, including social distancing, lockdown, and fear avoidance concerning the virus, might have contributed to these findings. This was expected, as several countries have recommended postponing non-urgent healthcare services, and as a consequence, several regions reported fewer non-urgent rehabilitation services as a response to the pandemic [15], and the same strategies were implemented in Saudi Arabia.

### 4.3. Therapy Services during the COVID-19 Lockdown

To help children with disabilities and their families adapt to their conditions, rehabilitation services should be organized and maintained as much as possible, according to the needs of each child or family. Thus, these services can be categorized into two categories: those needing telephone contact with family members and those requiring home visits for urgent cases [16].

In light of the COVID-19 pandemic, the current study claimed that 94% of caregivers did not receive any therapeutic services during the lockdown, with only a few receiving telephone advice and recording therapy sessions. The finding was in agreement with a study that showed that telehealth and online sessions substituted for the closure of rehabilitation centers [17]. The limited telehealth services noted in this study could be predicted, as telehealth is a comparatively recent mode of service delivery in Saudi Arabia and is mainly concentrated in urban centers [5].

Such interruptions in rehabilitation services could predictably deteriorate the communication, physical, and functional abilities of children. Thus, as shown by the results of this study, approximately 73% of the caregivers reported relapse in the conditions of their children because of the lockdown and cessation of therapy sessions, which is consistent with the findings of Cacioppo et al. [16]. The same finding was also reported by Boldrini et al. [18], who stated that the suspension of therapeutic sessions resulted in the deterioration of the skills and occupational performance of individuals with disabilities. Utilizing telehealth as a service delivery model for rehabilitation services is one potential means of overcoming the obstacles to receiving the services brought on by the pandemic, potential crises, a lack of rehabilitation centers, or restricted mobility.

Furthermore, 67% of the caregivers who participated in this study reported changes in their children’s moods during the lockdown. Children with disabilities are more likely to have mental health symptoms in normal living conditions [19], which could have been aggravated during the pandemic. The psychological effect of the lockdown is not limited to individuals with disabilities. Hence, upon reviewing the literature, it was found that the general population suffered from different psychological symptoms, including anxiety and anger related to the duration of the lockdown [20,21]. Caregivers of children with disabilities are particularly susceptible to these issues because their quality of life is already poor, even in the absence of a pandemic [22] Children with impairments often require assistance with daily activities, resulting in their parents experiencing physical strain, sleep deprivation, financial difficulties, and challenges in planning family vacations, all of which negatively impact their quality of life [23,24]. Furthermore, restrictions implanted during the COVID-19 pandemic have changed certain routines that were embedded in the lives of children with disabilities, and this may contribute to additional stress experienced by these families and the children [25], Lack of institutional assistance like schools, respite programs, and healthcare providers, as well as a lack of informal support from friends and family, may be particularly difficult for the caregivers [26]. In the current study, almost a third of the respondents reported that their children are not capable of carrying out daily activities; 35% of the children need some help from a person to do some activities of daily living; and 27% of them need help to do some activities of daily life.

The findings highlight the importance of providing continuous rehabilitation services to children with disabilities even during the lockdown, providing recommendations on the future of digital services and innovative methods for the delivery of rehabilitation services with proper training of therapists using telehealth, and offering proper education to caregivers on conducting home programs. Since telehealth technology is deemed important, efforts were recently introduced to establish collaborative guidelines by a group of experts in rehabilitation with participation from other industry professionals and key stakeholders. This will make it easier for rehabilitation clinicians working in Saudi Arabia to consult with or educate a patient remotely while monitoring them or delivering therapy or follow-up care [5,27]. 

The main limitation of the study involved the challenge of reaching a larger number of participants and the availability of participants as all rehabilitation centers were closed; therefore, the questionnaire was distributed online. Children with disabilities often have unique needs and require specialized services and care, which can limit their participation in research studies. This can result in a small pool of eligible participants, making it difficult to recruit a large sample size. In addition, data were collected using a self-administered questionnaire, which may have overestimated or underestimated the responses of the participants. The study design was cross-sectional, which limited the ability to show the cause-and-effect relationships of the variables. The study did not assess what services looked like before the pandemic (e.g., dose, length of time, service model: direct or consultative). The study was limited by the fact that we did not accurately measure the deterioration that occurs in children’s physical abilities, communication skills, and occupational performance after the pandemic. Additionally, this study did not determine the impact of the suspension of rehabilitation services on parents. Further studies need to be conducted using a qualitative approach to investigate the facilitators and barriers to telerehabilitation for children with disabilities.

## 5. Conclusions

The findings of this study revealed a drastic decline in the services provided to disabled children. The suspension of these services due to the pandemic has caused a notable deterioration in these children, as reported by their parents. This highlights the importance of developing recommendations to ensure the continued delivery of rehabilitation sessions even during pandemics through the enhancement of digital services, telerehabilitation, and home programs. Children with disabilities face numerous challenges in accessing rehabilitation services, which are essential for their physical, cognitive, and social development. Therefore, it is imperative to educate caregivers about their children’s rights to rehabilitation services through various media channels. One way to achieve this is by educating families about their children’s disabilities and providing them with the necessary skills to administer the intervention to their children in accordance with the guidelines provided by their therapists Thus, it seems that in such extreme conditions as a pandemic, telerehabilitation could limit the damage associated with the complete lack of access to rehabilitation services for disabled children. This reveals the importance of training all rehabilitation specialists on telerehabilitation and preparing an advance plan to ensure the optimal delivery of services.

## Figures and Tables

**Table 1:** Characteristics of the participants.

Variables	Frequency	Percent
**Child gender**		
Male	159	50.3
Female	157	49.7
**Child age categories (years)**		
1–3	68	21.5
4–6	93	29.4
7–9	61	19.3
10–12	46	14.6
13–15	22	7.0
16–18	26	8.2
**Diagnosis**		
Down’s syndrome	126	39.87
Cerebral palsy	120	37.97
Autism	53	16.77
Other genetic disorders	9	2.85
Other neurological disorders	8	2.53
**Impairment of the Child**		
Motor delay	20	6.3
Motor delay, mental delay	3	0.9
Motor delay, language delay	20	6.3
Motor delay, language delay, mental delay	107	33.9
Mental delay	40	12.7
Language delay	64	20.3
Speech delay, mental delay	62	19.6
**Number of siblings**		
1–5	271	85.77
6–10	43	13.6
>10	2	0.6
**Another child with a disability in the family**		
Yes	0	0
No	316	100
**Relationship of the caregiver**		
Father	84	26.6
Mother	199	63
Others	33	10.4
**Marital status**		
Married	297	94
Divorced	9	2.8
Widow	10	3.2
**Educational level of the caregiver**		
Primary education	16	5
University education	188	59.5
Intermediate education	79	25.0
Postgraduate degree	33	10.4

**Table 2:** Rehabilitation services received by children of the study group before the pandemic.

Items	Frequency	Percent
Does the child receive any therapy sessions? (speech/language, occupational therapy, physical therapy)		
Yes	280	88.6
No	36	11.4
Type of therapy		
Speech/Language	33	10.4
Speech/Language, Physiotherapy	8	2.5
Speech/Language, Occupational Therapy	68	21.5
Multidisciplinary rehabilitation (Speech/Language, Occupational Therapy, Physiotherapy)	160	50.6
Physiotherapy	14	4.4
Occupational Therapy	10	3.2
Physiotherapy, Occupational Therapy	23	7.3
Caregiver is a member of a support group with the same condition of child with disability		
Yes	162	51.3
No	154	48.7
Language level of the child		
child cannot speak	91	28.8
child speaks fluently	33	10.4
child can only form semi-sentences	80	25.3
child can only use simple words like bye-bye, milk, papa mama, etc.	112	35.4
Child’s self-reliance in performing activities of daily living		
child cannot carry out any activities of daily living	91	28.8
child is completely self-reliant in performing all activities of daily life	17	5.4
child needs some help from a person to do some activities of daily living	112	35.4
child can do some activities of daily life on his own, using some auxiliary tools and with the help of another person	85	26.9

**Table 3:** Subjects’ satisfaction with rehabilitation services before the pandemic.

Subjects’ Satisfaction	Median	Interpretation
Satisfaction of the parents with the speech/language therapy sessions provided for the child	4	Good
Satisfaction of the parents with the occupational therapy sessions provided to your child	4	Good
Satisfaction of the parents with the physical therapy sessions provided to your child	4	Good
Mother reported a continuous improvement after the speech and language sessions	4	Good
Mother reported a continuous improvement after the occupational therapy sessions	4	Good
Mother reported a continuous improvement of the child after the physiotherapy sessions	4	Good

**Table 4:** Consequences of the COVID-19 pandemic on therapy services.

Items	Frequency	Percentages
Child receive any private therapy sessions during the COVID-19 pandemic?		
Yes	19	6.0
No	297	94.0
Condition of the child deteriorated after staying at home for long periods and dropping out of sessions		
Yes	230	72.8
No	86	27.2
Experience difficulties adherence to the home speech and language therapy program during the lockdown		
Yes	12	63.2
No	7	36.8
Experience difficulties adherence to the occupational therapy home therapy program during the lockdown period?		
Yes	12	70.6
No	5	29.4
Experience difficulties adherence to the home exercise program during the lockdown period?		
Yes	12	66.7
No	6	33.3
Intend to return child to the rehabilitation sessions at the center or hospital		
When the lockdown ends	3	15.8
If the child’s condition is delayed more	3	15.8
When the COVID-19 pandemic is declared over	10	52.6
I will be satisfied with home sessions and go to the center infrequently	3	15.8

**Table 5:** Type of therapeutic service provided to the child during the COVID-19 pandemic.

Items	Frequency	Percentages
None	297	94.0
Telerehabilitation services	6	1.9
Therapy delivered by the caregiver	1	0.3
Home therapy sessions by the specialist	1	0.3
Remote therapy sessions via the internet	3	0.9
Recorded therapy sessions sent by the therapist	6	1.9
Therapy support groups via the social media	1	0.3
Therapy support groups via social media, mother applying exercises	1	0.3

**Table 6:** The frequency distribution and comparison of the accessibility of the services before and during the COVID-19 pandemic.

Accessibility of Services	Before COVID-19 Pandemic	During COVID-19 Pandemic	*p*-Value
Yes	280 (88.6%)	19 (6%)	0.001
No	36 (11.4%)	297 (94%)	

## Data Availability

The identified datasets analyzed during the current study are available from the corresponding author on reasonable request.

## Appendix A

**Table A1:** Comparison of the evaluation of the benefit of the support provided by the specialist during the lockdown period between different diagnoses.

Type of Therapy Support	DS (*n* = 8)	CP (*n* = 6)	Autism (*n* = 4)	Other NDs (*n* = 1)	*p*-Value
	Median	Median	Median	Median	
Speech-language	1.5	4	2.5	2	0.83
Occupational therapy	1.5	3.5	3.5	2	0.57
Physical therapy	2	3.5	3	2	0.93

DS—Down syndrome; CP—cerebral palsy; NDs—neurological disorders.
